# Biochemical and Thermodynamical Characterization of Glucose Oxidase, Invertase, and Alkaline Phosphatase Secreted by Antarctic Yeasts

**DOI:** 10.3389/fmolb.2017.00086

**Published:** 2017-12-12

**Authors:** Yassef Yuivar, Salvador Barahona, Jennifer Alcaíno, Víctor Cifuentes, Marcelo Baeza

**Affiliations:** Laboratorio de Genética, Departamento de Ciencias Ecológicas, Facultad de Ciencias, Universidad de Chile, Santiago, Chile

**Keywords:** antarctic yeasts, glucose oxidase, invertase, alkaline phosphatase, yeast secreted enzymes

## Abstract

The use of enzymes in diverse industries has increased substantially over past decades, creating a well-established and growing global market. Currently, the use of enzymes that work better at ambient or lower temperatures in order to decrease the temperatures of production processes is desirable. There is thus a continuous search for enzymes in cold environments, especially from microbial sources, with amylases, proteases, lipases and, cellulases being the most studied. Other enzymes, such as glucose oxidase (GOD), invertase (Inv), and alkaline phosphatase (ALP), also have a high potential for application, but have been much less studied in microorganisms living in cold-environments. In this work, secretion of these three enzymes by Antarctic yeast species was analyzed, and five, three, and five species were found to produce extracellular GOD, Inv, and ALP, respectively. The major producers of GOD, Inv, and ALP were *Goffeauzyma gastrica, Wickerhamomyces anomalus*, and *Dioszegia* sp., respectively, from which the enzymes were purified and characterized. Contrary to what was expected, the highest GOD and Inv activities were found at 64°C and 60°C, respectively, and at 47°C for ALP. However, the three enzymes maintained a significant percentage of activity at lower temperatures, especially ALP that kept a 67 and 43% of activity at 10°C and 4°C, respectively.

## Introduction

In the past decades, the use of enzymes in diverse industries such as food, detergent, paper, textile, synthesis of organic compounds, etc., has increased substantially, because they are environmentally friendly and highly efficient. At present, industrial/biotechnological enzymes represent a well-established global market projected to reach US$6.3 billion in 2021 (Polaina and MacCabe, 2007; Shahani Dewan, 2017). Currently, it is desirable that enzymes used in production processes have high activity at ambient or lower temperatures, in order to save energy in large-scale processes and to easily inactivate them through selective thermal inactivation when it is required (Sarmiento et al., 2015). For this reason, there is a continuous search for enzymes having better performance at low temperatures, especially from microbial sources thriving in cold environments. The most studied cold-active enzymes are those used in the food, textile, biofuel, and brewing industries (Białkowska and Turkiewicz, 2014), such as amylases, proteases, lipases, and cellulases (Kuddus and Roohi, 2010; Joshi and Satyanarayana, 2013; Maiangwa et al., 2015). The enzymes glucose oxidase (GOD), invertase (Inv), and alkaline phosphatase (ALP) have promising applications in diverse fields such as the food, medical, and pharmaceutical industries; however, these enzymes have been much less studied in microorganisms living in cold environments. GOD (β-D-glucose:oxygen 1-oxidoreductase; EC 1.1.2.3.4) catalyzes the oxidation of β-D-glucose to gluconic acid, also producing hydrogen peroxide, by using molecular oxygen as an electron acceptor (Bankar et al., 2009). This enzyme is applied in the food, beverage, chemical, pharmaceutical, and biotechnological industries (Ferri et al., 2011; Du et al., 2013; Dubey et al., 2017), and has novel applications in biosensors (Bankar et al., 2009; Wang et al., 2013). Due to this potential, the search for GODs from microbial sources is increasing. The most studied and commercialized GOD was obtained from the fungus *Aspergillus niger*; it has a high substrate specificity and is stable over a wide range of pH and temperature (Bankar et al., 2009; Sumaiya and Trivedi, 2015). Inv (β-D-fructofuranoside fructohydrolase; EC 3.2.1.26) hydrolyzes the β 1-2 linkage of sucrose, producing glucose and fructose as products, and it is used in the manufacture of artificial honey, enzyme electrodes for the detection of sucrose, and in the pharmaceutical industry (Kulshrestha et al., 2013; Kadowaki et al., 2014). This enzyme is widely distributed throughout the biosphere, and it has been characterized mainly in plants and microorganisms. ALP (EC 3.1.3.1) is a phospho-monoesterase that catalyzes the hydrolysis of phosphoric acid to alcohol and phosphorus at alkaline pH, playing multiple biological functions in organisms from bacteria to mammals; it is used in medicine, pharmacology, and agriculture (Rankin et al., 2010; Nalini et al., 2015). The ALPs most studied have been obtained from mammals (bovine) and *Escherichia coli*, having higher activity over the temperature range 27–37°C and at pH 8.0–12.0 (Tuleva et al., 1998; Rankin et al., 2010).

Yeasts inhabiting cold-environments have attracted much attention as their hydrolytic enzymes, in general, have a higher activity at lower temperatures than those from template environments. In addition, these enzymes may be secreted by yeasts, facilitating large-scale production, which is another property desirable for biotechnological and industrial applications (Buzzini and Margesin, 2014; Alcaíno et al., 2015). In this work, diverse species of yeasts isolated from King George Island, in the sub-Antarctic region (Carrasco et al., 2012) were evaluated for the production of extracellular GOD, Inv, and ALP enzymes, which were found in five, three, and five yeast species, respectively. The enzymes secreted by *G. gastrica, W. anomalus*, and *Dioszegia* sp. were purified, and biochemically and thermodynamically characterized.

## Methods

### Yeast strains and culture conditions

The yeasts used in this study were originally isolated from soil samples from King George island, the major island of the South Shetland Archipelago (sub-Antarctic region) (Carrasco et al., 2012), and are listed in Table 1. Yeasts were grown in YM medium (0.3% yeast extract, 0.3% malt extract, 0.5% Bacto peptone) supplemented with 1% glucose for GOD and ALP studies, and with 1% sucrose for Inv studies. The incubation temperature corresponded to that for the optimal growth of each yeast species (Table 1).

**Table 1:** Yeasts species used in this study.

**Species**	**Temp.^a^**	**Protein^b^**
*Candida sake*	22	75
*Cryptococcus gastricus* (*Goffeauzyma gastrica*)	22	90
*Cryptococcus* (*Goffeauzyma*) *gilvescens*	22	65
*Cryptococcus* (*Vishniacozyma*) *victoriae*	22	80
*Dioszegia* sp.	15	99
*Glaciozyma antarctica*	10	73
*Leucosporidiella creatinivora* (*Leucosporidium creatinivorum*)	22	80
*Leucosporidiella fragaria* (*Leucosporidium fragarium*)	22	49
*Mrakia psychrophila*	10	42
*Metschnikowia bicuspidata*.	10	41
*Mrakia* sp.	15	54
*Rhodotorula* (*Phenoliferia*) *glacialis (T11Rs)*	15	80
*Rhodotorula* (*Cystobasidium*) *laryngis*	22	61
*Sporidiobolus salmonicolor*	22	60
*Wickerhamomyces anomalus*	30	113

*In parenthesis is indicated the current taxonomic classification for genera and species, as used in the text, are indicated in parentheses*.

a *Optimum temperature for growth*.

b *Protein concentration of protein samples (μg/ml)*.

### Extracellular protein extraction

Yeast cultures at the early stationary phase of growth (100 ml) were centrifuged at 6,000 × g for 10 min at 4°C, and the supernatant was filtered through a polyvinylidene fluoride membrane of 0.45 mm pore-size (Millipore, Billerica, MA, USA). Proteins in the cell-free supernatant were precipitated by adding ammonium sulfate at 80% saturation, followed by incubation at 4°C for 2 h with constant agitation and centrifugation for 15 min at 10,000 × g at 4°C. The pellet was suspended in 4 ml of Tris-HCl buffer (0.1 M, pH 7.0) and dialyzed in tubes with 10 kDa molecular weight cut-off against 1,000 ml of Tris-HCl buffer (0.1 M, pH 7.0) for 12 h at 4°C, changing the buffer two to three times. Proteins were stored at −20°C until analysis. The protein content in samples was quantified using a BCA Kit Assay (Thermo Scientific, IL, USA) according to the manufacturer's instructions, and visualized by sodium dodecyl sulfate polyacrylamide gel electrophoresis (SDS-PAGE). The protein Mr was estimated from SDS-PAGE gels using the PageRuler Plus Prestained Protein Ladder (Thermo Scientific, IL, USA) as a commercial protein reference standard.

### Determination of enzyme activities

To determine GOD activity, 50 μl of each protein sample was mixed with 50 μl of phosphate-citrate buffer with 10% glucose and incubated for 1 h. The reaction was then mixed with 98 μl of 0.2 M phosphate buffer, 0.17 mM o-dianisidine, and 2 μl peroxidase (6 U ml^−1^), incubated at 25°C for 30 min, and the absorbance at 500 nm was determined. To measure Inv activity, 50 μl of protein sample was mixed with 50 μl of a solution containing phosphate-citrate buffer and 0.2 M sucrose, and incubated for 1 h. The release of reducing sugars was quantified by the DNS method (Miller, 1959). The ALP activity was determined using an Alkaline Phosphatase Assay kit (Abnova, Taipei, Taiwan) according to the manufacturer's instructions. The temperature and pH in the enzyme assays varied from 4 to 84°C and from pH 2.6 to 11.5, respectively.

### Enzyme purification and analysis

Proteins from cell-free supernatants were differentially precipitated by adding ammonium sulfate at increasing saturation (20–80%). In each precipitation step, the sample was incubated at 4°C for 2 h and centrifuged at 10,000 × g at 4°C for 15 min. Protein pellets were suspended in 2 ml of potassium phosphate buffer (20 mM and pH 7.0) and desalted using a HiTrap Desalting column (GE, Schenectady, New York, USA). Proteins were separated using molecular exclusion or anion exchange chromatography. For molecular exclusion chromatography, Superdex 75 10/300 GL and Superdex 200 10/300 GL columns were used with a mobile phase of 50 mM phosphate, 150 mM NaCl, pH 7.0, at 0.2 ml min^−1^ flow rate. For anion exchange chromatography, a HiPrep 16/10 QFF column was used with a mobile phase of 20 mM Tris-HCl, pH 8.0, and a NaCl gradient from 0 to 1 M at 0.5 ml min^−1^ flow rate. The columns were operated using ÄKTAprime plus equipment (General Electrics, New York, USA).

### Kinetics and thermodynamic determinations

To evaluate several physical-chemical parameters on enzyme activities, a two-level Plackett-Burman design was applied as detailed in Table S1. In each assay, the progression of the enzyme reaction was determined and the calculated specific reaction rates were used to determine the effect of each factor. Then, taking into account the two main factors that affected their activity, the conditions for highest activity of each enzyme were determined using a central composite design, and used for the determination of kinetic parameters. The initial reaction velocities at different substrate concentrations were calculated, and *V*_max_ and *K*_m_ were calculated according to the Michaelis-Menten model. To evaluate the irreversible thermal inactivation of enzymes, their activities were determined at temperatures from 4 to 84°C, incubating for 1 to 8 h. The inactivation rate constant (*k*) at different temperatures was obtained from the slope of plots of the natural logarithm of percentage of activity vs. the reciprocal of the absolute temperature. The activation energy (*E*_*a*_) was obtained from the slope of the plot of the natural logarithm of *k* vs. the reciprocal of the absolute temperature. The amount of GOD, Inv and ALP used in the assays were 15.5 ug, 39.0 ug and 39.0 ug, respectively, in 100 μl final volume of the reaction. The concentration of glucose varied from 0.006 to 11 mM, of sucrose from 1 to 100 mM and of p-Npp from 0.001 to 2 mM.

## Results

### Screening extracellular enzyme activity

**Figure 1:**
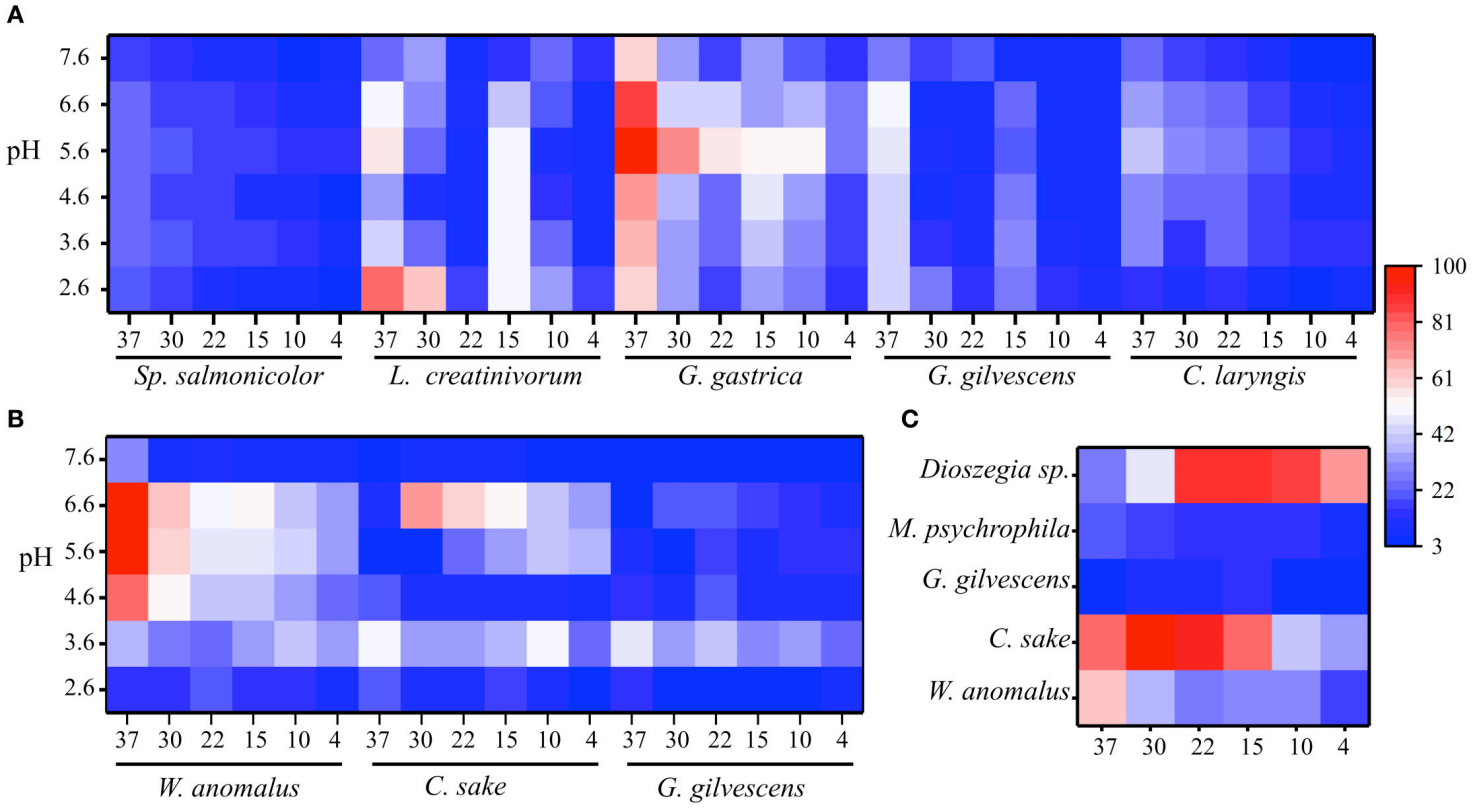
Enzyme activities at different temperature and pH. Enzyme activity levels were determined as the change of absorbance at 500 nm for GOD **(A)**, 540 nm for Inv **(B)**, and 405 nm for ALP **(C)** and normalized by total protein amount in each sample (indicated in Table 1). In each case, the percentages were calculated considering as 100% the highest activity that was measured under each condition among yeasts species. In each heat-map, the x-axis is the temperature (Supporting data in Table S2). The substrate concentration used was 280, 100, or 5 mM of glucose, sucrose or pNPP.

The extracellular protein samples obtained from cultures of different yeast species were evaluated for GOD, Inv, and ALP activities at several temperatures and pH values. The results obtained from samples that displayed at least one enzyme activity are shown in Figure 1. GOD activity was detected in protein samples from five yeast species and, in general, the highest activity was detected at 30 to 37°C and pH 5.6 to 6.6 (Figure 1A). The exception was the protein sample from *Leucosporidium creatinivorum*, which displayed the maximum GOD activity at pH 2.6. The highest GOD activity was found in the protein sample from *Goffeauzyma gastrica* at 37°C and pH 5.6. Protein samples from three yeast species displayed Inv activity, which was highest at 30–37°C and pH 3.6–6.6. The highest Inv activity was detected in the protein sample from *Wickerhamomyces anomalus* at 37°C and pH 5.6 to 6.6 (Figure 1B). ALP activity was assayed at pH 10.0 and was detected in protein samples from *W. anomalus, Candida sake*, and *Mrakia psychrophila* with a maximum activity at 30 to 37°C. *Goffeauzyma gilvescens* and *Dioszegia* sp. displayed the highest ALP activity at 15°C and ≤ 22°C, respectively (Figure 1C).

As the protein samples from *G. gastrica* and *W. anomalus* displayed the highest GOD and Inv activity, respectively, these species were selected for further analyses to characterize the respective enzymes. The highest ALP activity was observed in samples from *C. sake* and *Dioszegia* sp.; the latter was selected for additional studies because it showed the highest enzyme activity at a lower temperature (15°C).

### Enzyme purification and effect of physical-chemical factors on enzyme activity

**Figure 2:**
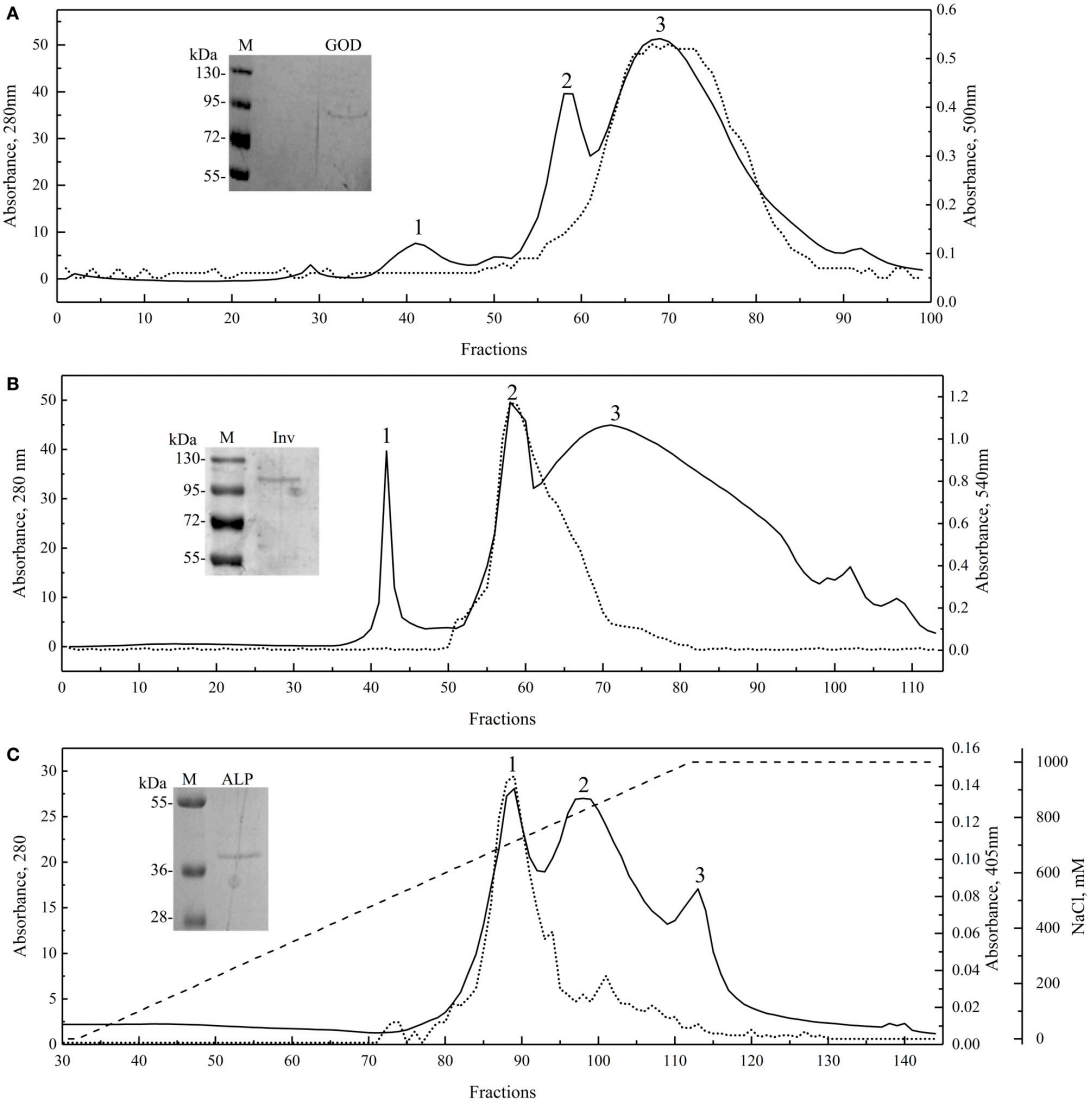
Enzyme purification. Molecular exclusion chromatograms for GOD (**A**; Superdex 75 10/300 GL column) and Inv (**B**; Superdex 200 10/300 GL column), and anion exchange chromatogram for ALP (**C**; HiPrep 16/10 QFF column; NaCl gradient shown as discontinuous line). Runs were recorded at 280 nm, and each fraction was evaluated for enzyme activity (dotted lines). SDS-PAGE of the purified and concentrated enzyme final sample are shown in each chromatogram.

Proteins from yeast culture supernatants were fractioned by precipitation with ammonium sulfate from 20 to 80% saturation; each fraction was analyzed for protein content and enzyme activity. As shown in Figure S1, the Inv and ALP activities were detected in the 80% ammonium sulfate fraction of protein samples from *W. anomalus* and *Dioszegia* sp., respectively, while GOD activity was detected in the 60% ammonium sulfate fraction of the *G. gastrica* sample. However, multiple protein bands were observed in fractions having enzyme activity, so these fractions were subjected to additional purification steps by exclusion or ionic interchange chromatography (Figure 2). Three main peaks were observed in all chromatograms, and in each particular case, peaks 3, 2, and 1, coincided with the fractions having GOD, Inv, and ALP activity, respectively. Fractions in which the enzyme activity was detected and had a unique protein band were pooled together and concentrated in order to obtain the purified sample of each enzyme (Figure 2). According to the SDS-PAGE analysis, the GOD, Inv, and ALP activity would correspond to a protein band of Mr 91,000, 101,000, and 39,000, respectively. The purified enzyme samples were used to determine the physical-chemical factors that mainly influence the activity of each enzyme. The temperature and pH were tested for all enzymes, and the concentration of glucose, FeSO_4_, and CaCl_2_ was tested for GOD, the concentration of H_2_O_2_, urea, and ethanol was tested for Inv, and the concentration of ZnS0_4_, MgCl_2_, and L-phenylalanine was tested for ALP. According to this analysis, pH and temperature were the second most important factors affecting the activity of the three studied enzymes (under the ranges tested), with the addition of CaCl_2_, ethanol, and MgCl_2_ being the main factors affecting GOD, Inv, and ALP, respectively (Table S1). However, by analyzing the results of each assay, enzyme activity only increased in the case of adding CaCl_2_, contrary to the addition of ethanol or MgCl_2_ in which cases the enzyme activity decreased.

### Determination of conditions for highest enzyme activity and biochemical parameters

**Figure 3:**
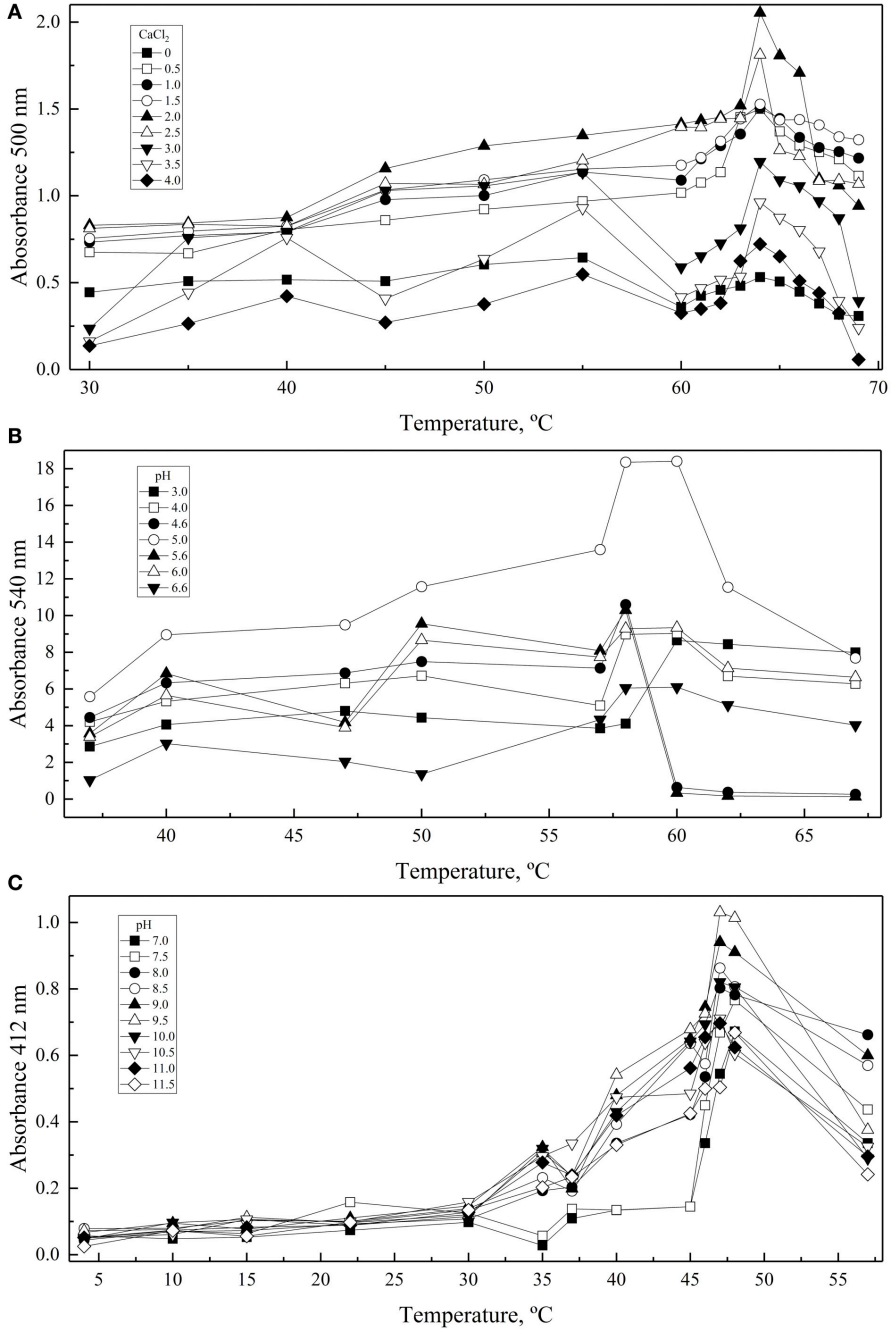
Effect of physical-chemical factors on enzyme activity. Enzymatic reactions were conducted varying the CaCl_2_, pH, and temperature in each central composite design: CaCl_2_ and temperature for GOD **(A)**, and pH and temperature for Inv **(B)** and ALP **(C)**. Each point corresponds to the maximum values of absorbance obtained from progress curves performed at each condition. Total amounts of enzyme used in each assay were 15, 39, or 39 ug of GOD, Inv or ALP, respectively. The substrate concentration used was 280, 100, or 5 mM of glucose, sucrose or pNPP.

A central composite design with two levels was applied to determine the conditions at which each enzyme had the highest activity considering the two-main physical-chemical factors that affected the activity of each one. GOD showed the highest activity at 64°C and with 2 M CaCl_2_, Inv at 60°C and pH 5.0, and ALP at 47°C and pH 9.0 (Figure 3). These conditions were used to determine the enzymes' kinetic parameters using glucose, sucrose, and *p*-nitrophenylphosphate at different concentrations as substrates for GOD, Inv, and ALP, respectively. All enzymes displayed a Michaelis-Menten kinetic (Figure S2) and the calculated parameters are listed in Table 2. The calculated catalytic efficiency (*k*_cat_/*K*_M_) for GOD, Inv, and ALP was 1.4 × 10^7^ M^−1^ s^−1^, 4.3 × 10^7^ M^−1^ s^−1^, and 2.4 × 10^6^ M^−1^ s^−1^, respectively, indicating that these enzymes are highly efficient.

**Table 2:** Kinetic and thermodynamic parameters of enzymes secreted by Antarctic yeasts.

	**GOD^a^**	**Inv^b^**	**ALP^c^**
*V*_max_ (mM min^−1^)	0.46	99.5	0.11
*K*_m_ (mM)	1.6	78.6	0.59
*K*_cat_ (s^−1^)	2.3 × 10^4^	4.4 × 10^6^	1.4 × 10^3^
*E*_a_ (kJ mol^−1^)	35.1	25.2	8.2

a *From G. gastrica*.

b *From W. anomalus*.

c *From Dioszegia sp*.

*The total amount of enzyme used in each assay was 15, 39, and 39 ug of GOD, Inv and ALP, respectively*.

**Figure 4:**
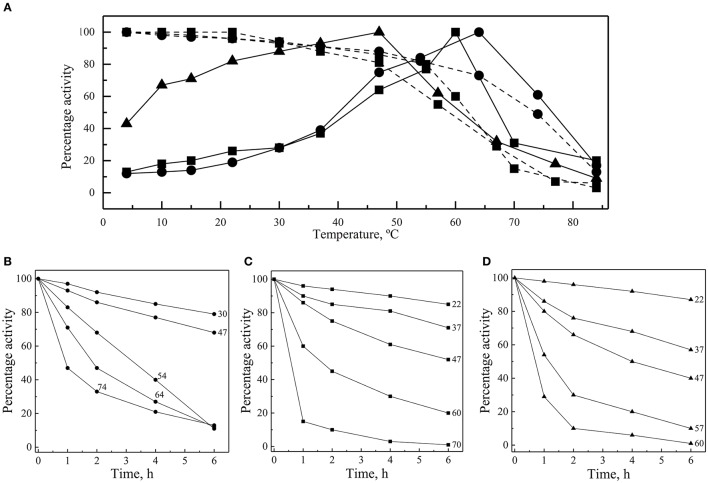
Thermal effect on activity and stability of enzymes. **(A)** Enzyme activities of GOD (circles), Inv (squares) and ALP (triangles) were measured at different temperatures (continuous lines). Enzyme samples were incubated for 1 h at different temperatures and then activities were determined at 64°C, 60°C, and 47°C for GOD, Inv and ALP, respectively (discontinuous lines). Activity was also measured after incubation at different temperatures for different times, and the relative enzyme activity, compared with the maximum activity reached in each case, is shown for GOD **(B)**, Inv **(C)**, and ALP **(D)**. The total amount of enzyme used in each assay was 15, 39, or 39 ug of GOD, Inv or ALP, respectively; the substrate concentration used was 280, 100, or 5 mM of glucose, sucrose or pNPP, respectively. The Inv and ALP activity assays were performed at pH 5.0 and pH 9.0, respectively, and GOD activity determination was made in presence of 2 M CaCl_2_.

The thermal effect on the enzymes' activity was assayed at 4 to 84°C, considering as 100% the activity of each enzyme at its optimal temperature. In all cases, the greatest activity decay was observed at higher rather than at lower temperatures (Figure 4A). At 30°C, the percentage of GOD and Inv activity was 28%, while it was 88% for ALP. ALP was the enzyme that displayed the highest percentage of activity at lower temperatures with 67% at 10°C, while the percentages of GOD and Inv activities were 13% and 18%, respectively, at the same temperature. At 4°C, the percentage of ALP activity was 43%.

**Figure 5:**
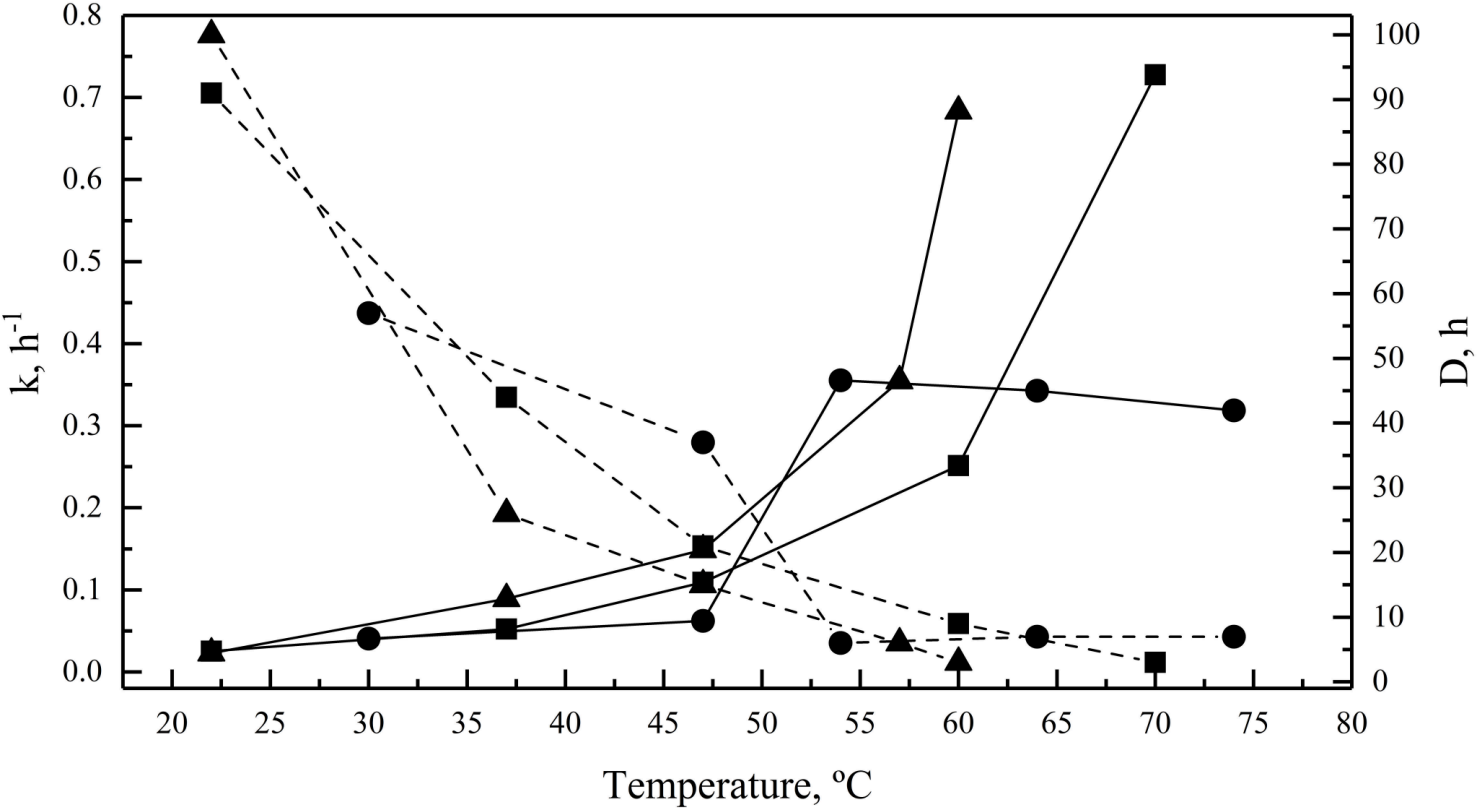
Enzyme thermal inactivation. The inactivation rate constant (*k*, continuous lines) and *D*-values (discontinuous lines) are shown for GOD (circles), Inv (squares), and ALP (triangles). The total amount of enzyme used in each assay was 15, 39, or 39 ug of GOD, Inv or ALP, respectively; the substrate concentration used was 280, 100, or 5 mM of glucose, sucrose or pNPP, respectively.

Enzymes were also incubated for 1 h at different temperatures before performing the enzymatic assay at their optimal temperatures for activity. All of them retained at least 80% activity when they were previously incubated at temperature ranging from 4 to 54°C, 4 to 55°C, and 4 to 47°C for GOD, Inv, and ALP, respectively (Figure 4A). The percentage of activity strongly declined after 1 h of incubation at higher temperatures. In incubations for longer times, the GOD activity decayed linearly across 30, 47, and 54°C, maintaining 79, 68, and 11% of activity, respectively, after 6 h of incubation (Figure 4B). After 6 h of incubation, the percentage of Inv activity was 85, 71, and 52% when incubated at 22, 37, and 47°C, respectively, displaying a faster decay at 60 and 70°C (Figure 4C). Similarly, at 22, 37, and 47°C, ALP retained 87, 57, and 40% of activity, respectively, after 6 h of incubation, which declined faster at 57 and 60°C (Figure 4D). The calculated activation energy from the Arrhenius plots (Figure S3) was 35, 25, and 8 kJ mol^−1^ for GOD, Inv, and ALP, respectively. The enzyme inactivation rates increased as the temperature increased, as indicated by the increment of the inactivation constant (*k*), and, accordingly, the time required to reduce the enzyme activity to 10% (*D*-value) decreased as the temperature increased (Figure 5).

## Discussion

The three enzymes studied in this work have applications in several industries: GOD in food, beverage, and textile industries, and in glucose biosensors (Bankar et al., 2009; Dubey et al., 2017); Inv in the production of invert sugars and prebiotic compounds (Kulshrestha et al., 2013; Kadowaki et al., 2014; Nadeem et al., 2015); and ALP in diagnostic studies, in biosensors and as a marker for milk pasteurization (Rankin et al., 2010; Nalini et al., 2015). To the best of our knowledge, there are not many studies (or even none) regarding the secretion of GOD, Inv, and ALP enzymes in yeasts, especially from yeasts inhabiting cold environments. In this work, the secretion of these enzymes was found in several Antarctic yeast species, which were analyzed expecting to find enzyme activity at lower temperatures. The GOD secreted by *G. gastrica* described in this work displayed highest activity at 60°C, similar to GOD described from *Aspergillus tubingensis* and a recombinant GOD from *Penicillium amagasakiense* (Courjean and Mano, 2011; Kriaa et al., 2015), but higher to those described in *A. niger, P. amagasakiense*, and *Penicillium* sp. CBS 120262, and a recombinant GOD was produced in *Yarrowia lipolytica*, which had higher activity at temperatures from 25 to 40°C (Kalisz et al., 1997; Bhatti et al., 2006; Simpson et al., 2007; Khadivi Derakshan et al., 2017). The *K*_M_ of the GOD from *G. gastrica* (1.6 mM) was lower than that described in GODs from filamentous fungi such as *Talaromyces flavus, A. niger*, and *Penicillium* species (Karmali et al., 2004; Bhatti et al., 2006; Eremin et al., 2006; Simpson et al., 2007; Bankar et al., 2009), and a recombinant one expressed in *Pichia pastoris* and immobilized, whose *K*_M_ ranged from 5.7 to 33.0 mM (Courjean and Mano, 2011; Otadi and Mobayen, 2011; Kovačević et al., 2014).

To the best of our knowledge, the only Inv described from a yeast isolated from Antarctica, was from *Leucosporidium antarcticum* and was an intracellular enzyme that showed its highest activity at 30°C and pH 4.6 to 4.8 (Turkiewicz et al., 2005). The extracellular Inv purified in this work from Antarctic *W. anomalus* showed its best activity at 60°C and pH 5.0, which is in the range of temperature (30 to 80°C) and pH (2.0 to 6.5) reported for Inv enzymes in other yeasts such as *Xanthophyllomyces dendrorhous, Rhodotorula glutinis, Schizosaccharomyces pombe, Saccharomyces cerevisiae, Candida utilis, Kluyveromyces marxianus*, and *Rhodotorula dairenensis* (Acosta et al., 2000; Belcarz et al., 2002; Rubio et al., 2002; Gutiérrez-Alonso et al., 2009; Linde et al., 2009; Aziz et al., 2011; Aburigal et al., 2014; Shankar et al., 2014); and in the filamentous fungi *Paecylomices variotii, Aspergillus flavus*, and *Aspergillus terreus* (Uma et al., 2010; Giraldo et al., 2012; Shaker, 2015). In these fungi, the *K*_M_ values described for their Inv were from 1.5 to 227.0 mM, range in which is the *K*_M_ of the Inv of *W. anomalus* (78.6 mM), described in this work.

The ALP from *Dioszegia* sp. purified in this work showed the highest activity at 47°C and pH 9.0, similar to those described from plants and mammals (35 to 45°C and pH 9.0 to 10.0) (Njoku et al., 2011; Swargiary et al., 2013), and from other microbial sources (40 to 70°C and pH 9.5 to 11.0) (Hauksson et al., 2000; Zappa et al., 2001; Golotin et al., 2015). The *K*_M_ of the ALP described in this work was 0.59 mM, which is the range of *K*_M_ values described from other organisms such as the from rabbit *Lepus townsendii* liver (0.5 mM) (Njoku et al., 2011), from *Vibrio* sp. (0.11 mM) (Asgeirsson et al., 2007) and a recombinant ALP from marine bacterium *Cobetia marina* (13.2 mM) (Golotin et al., 2015).

As previously mentioned, the Antarctic yeast species that were studied in this work were under the premise that the enzymes produced by them would be active at low temperatures; however, the GOD and Inv enzymes showed the highest activity at temperatures above 60°C. Nevertheless, these enzymes kept a significant activity at lower temperatures: GOD, 75% and 40% at 47°C and 37°C, respectively, and Inv, 64 and 40% of activity at 47°C and 37°C, respectively. The best results were obtained for ALP, enzyme that showed the highest activity at 47°C and kept a high activity at minor temperatures: over 80% at 22°C, and outstandingly, a 67 and 43% of activity at 10°C and 4°C, respectively.

An important aspect to be considered for the production of enzymes is the current interest and preference of the use of microorganisms due to their characteristics that support the economic feasibility of the production. Among them, rapid growth using inexpensive media, high production yields, a regular supply due to the absence of seasonal fluctuations, an easier product modification, the possibility of optimization and to perform genetic manipulations to increase the enzyme production, to mention some (Anbu et al., 2013; Gurung et al., 2013; Adrio and Demain, 2014). Considering the biochemical and thermodynamic properties of the GOD, Inv, and ALP enzymes studied in this work, these enzymes are interesting candidates to perform further studies to evaluate their potential application in productive areas. Furthermore, these enzymes are secreted by the yeasts that produced them, a characteristic that would simplify the enzyme purification step necessary for their use at an industrial scale.

## Author contributions

MB conceived the study; YY performed the majority of the experiments; SB participated in protein extractions and analysis; JA and MB participated in the experimental design and results analyses. MB, YY, JA, and VC drafted the manuscript. All authors read and approved the final manuscript.

### Conflict of interest statement

The authors declare that the research was conducted in the absence of any commercial or financial relationships that could be construed as a potential conflict of interest.

## Supplementary material

Figure S1 Protein sample fractioning and enzyme activity determination. Total protein samples from *G. gastrica* **(A)**, *W. anomalus* **(B)**, and *Dioszegia* sp. **(C)** were fractioned by ammonium sulfate precipitation at 20% (lanes 1, 5, and 9), 40% (lanes 2, 6, and 10), 60% (lanes 3, 7, and 11) and 80% (lanes 4, 8, and 12) saturation. Each fraction was analyzed by SDS-PAGE, and the GOD **(A)**, Inv **(B)**, and ALP **(C)** activities were determined. At the bottom of the gels, the presence (+) or absence (−) of the respective activity is indicated.

Figure S2 Reaction rate in relation to substrate concentration. **(A)**, GOD; **(B)**, Inv; **(C)** ALP. The total amount of enzyme used in each assay was 15, 39, or 39 ug of GOD, Inv, or ALP, respectively.

Figure S3 Thermal inactivation of enzymes. Arrhenius plots for GOD (circles), Inv (squares), and ALP (triangles) are shown. The total amount of enzyme used in each assay was 15, 39, or 39 ug of GOD, Inv, or ALP, respectively; the substrate concentration used was 280, 100, or 5 mM of glucose, sucrose or pNPP, respectively.

Table S1 Plackett-Burman design and calculation of the effect of physical-chemical parameters on enzyme activity.

Table S2 Enzyme activities at different temperature and pH. Enzyme activity levels were determined as the change of absorbance at 500 nm for GOD, 540 nm for Inv, and 405 nm for ALP and normalized by total protein amount in each sample. The substrate concentration used was 280, 100 or 5mM of glucose, sucrose or pNPP.

## Abbreviations

GOD: glucose oxidase
Inv: invertase
ALP: alkaline phosphatase
*K*_M_: Michaelis-Menten constant
V_max_: maximum velocity
*k*_cat_: catalytic constant
pNPP: p-nitrophenyl phosphate.
