# Supplementary figures and images for: Biochemical and Thermodynamical Characterization of Glucose Oxidase, Invertase, and Alkaline Phosphatase Secreted by Antarctic Yeasts
Source: Front Mol Biosci. 2017 Dec 12;4:86. doi: 10.3389/fmolb.2017.00086 (PMC5733001; doi:10.3389/fmolb.2017.00086)

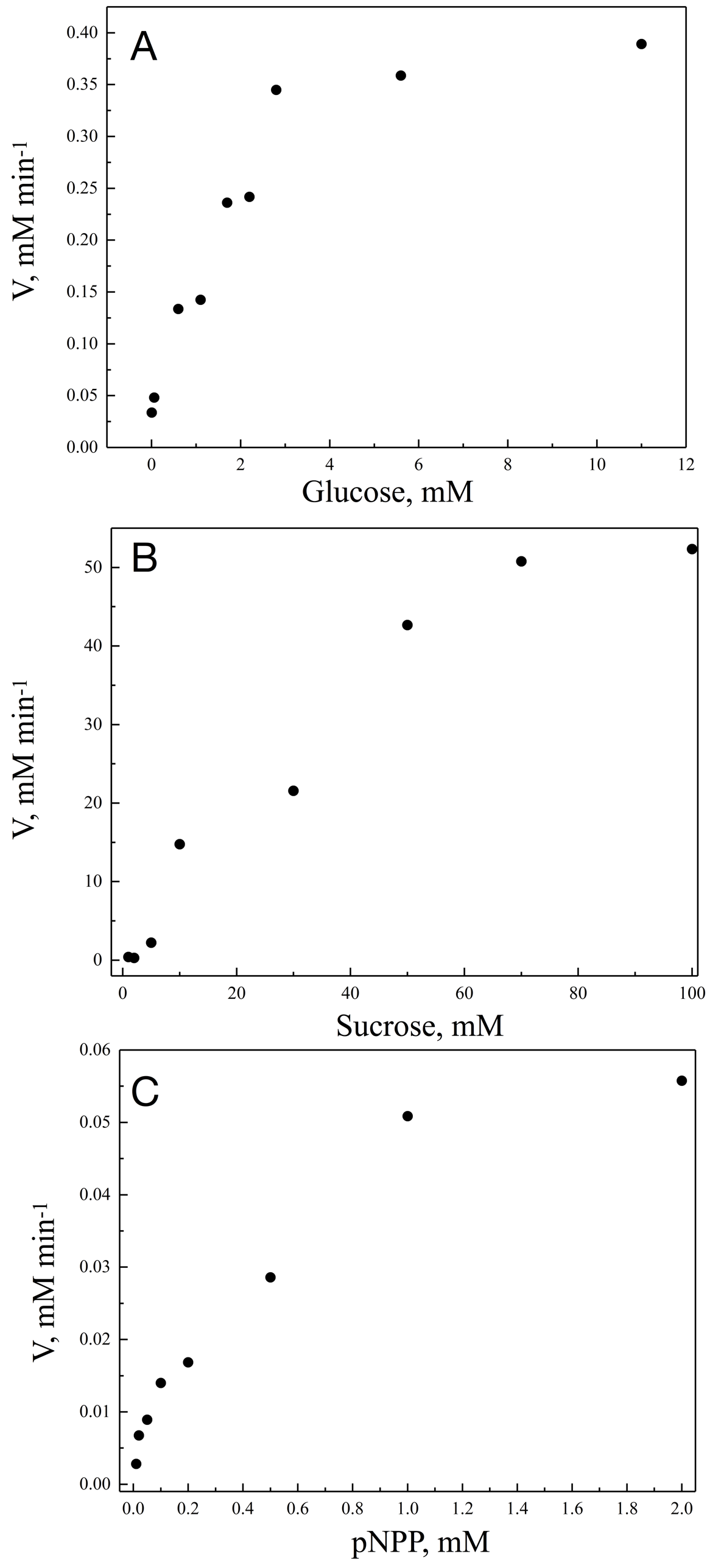

Supplement: Figure S2 — Reaction rate in relation to substrate concentration. (A), GOD; (B), Inv; (C) ALP. The total amount of enzyme used in each assay was 15, 39, or 39 ug of GOD, Inv, or ALP, respectively. [file Image2.JPEG]

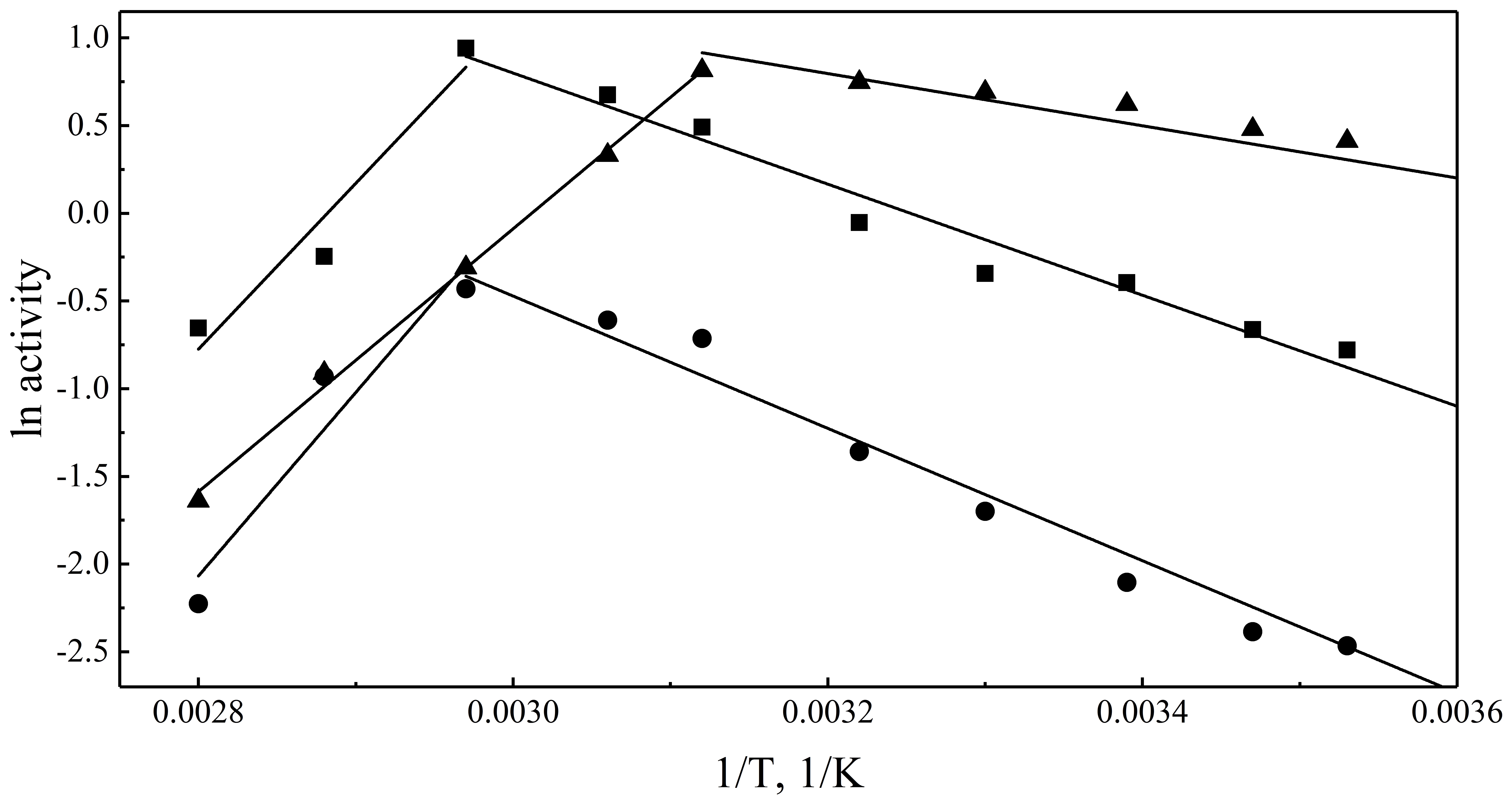

Supplement: Figure S3 — Thermal inactivation of enzymes. Arrhenius plots for GOD (circles), Inv (squares), and ALP (triangles) are shown. The total amount of enzyme used in each assay was 15, 39, or 39 ug of GOD, Inv, or ALP, respectively; the substrate concentration used was 280, 100, or 5 mM of glucose, sucrose or pNPP, respectively. [file Image3.JPEG]
