# Supplementary material for: Biochemical and Thermodynamical Characterization of Glucose Oxidase, Invertase, and Alkaline Phosphatase Secreted by Antarctic Yeasts
Source: Front Mol Biosci. 2017 Dec 12;4:86. doi: 10.3389/fmolb.2017.00086 (PMC5733001; doi:10.3389/fmolb.2017.00086)
Supplement: Table S1 — Plackett-Burman design and calculation of the effect of physical-chemical parameters on enzyme activity. [file Table1.PDF]

Table S1. Plackett-Burman design and calculation of the effect of physical-chemical parameters on enzyme activity.

Plackett-Burman desing								Rate (nn min^{-1})		
Assay	V1	V2	V3	V4	SV1	V5	SV2	GOD	Inv	ALP
1	+	+	+	-	+	-	-	2.80	0.19	0.20
2	-	+	+	+	-	+	-	11.90	0.33	0.02
3	-	-	+	+	+	-	+	2.35	0.66	0.03
4	+	-	-	+	+	+	-	0.60	0.05	0.05
5	-	+	-	-	+	+	+	6.35	0.07	0.13
6	+	-	+	-	-	+	+	3.80	0.13	0.06
7	+	+	-	+	-	-	+	0.80	0.23	0.06
8	-	-	-	-	-	-	-	0.50	0.75	0.06

Factors and effect on each enzyme activity												
	GOD				Inv				ALP			
	Factor	-	+	E	Factor	-	+	E	Factor	-	+	E
V1	pH	4.6	6.6	6.3	pH	4.6	6.6	5.9	pH	8	10	0.4
V2	Temp. (ºC)	27	47	6.3	Temp. (ºC)	27	47	5.9	Temp. (ºC)	4	22	0.4
V3	Glucose (%)	1	20	5,8	H_2O_2 (%)	0	0.1	0.2	$ZnS0_4$ (mM)	0	5	0
V4	$FeSO_4$ (mM)	0	0.2	0.2	Urea (M)	0	0.4	0.1	$MgCl_2$ (mM)	0	5	1.9
V5	$CaCl_2$ (M)	0	1	9.7	Ethanol (%)	0	8	6.3	L-phenylalanine	0	10	0.2

PB, Plackett-Burman design. V, variable; SV1 and SV2, simulated variables; -, low value; +, high value. E, effect of each variable calculated according too Placket-burmant design.

*, the physical-chemical parameters and the ranges evaluated for each enzyme activity are indicated. - and +, mean low and high values for each variable. 0 values means no addition of respective compounds. For each assay de progress curve was determined, follow the product formation by GOD, Inv and ALP, by the absorbance at 500 nm, 540 nm and 405 nm, respectively. Rate was calculated from initial period in each case.
